# Regulatory Function of a Novel Population of Mouse Autoantigen-Specific Foxp3^−^ Regulatory T Cells Depends on IFN-γ, NO, and Contact with Target Cells

**DOI:** 10.1371/journal.pone.0007863

**Published:** 2009-11-17

**Authors:** Cyndi Chen, Chih-Pin Liu

**Affiliations:** 1 Department of Immunology, Beckman Research Institute, City of Hope, Duarte, California, United States of America; 2 Department of Diabetes, Endocrinology, and Metabolism, Beckman Research Institute, City of Hope, Duarte, California, United States of America; New York University School of Medicine, United States of America

## Abstract

**Background:**

Both naturally arising Foxp3^+^ and antigen-induced Foxp3^−^ regulatory T cells (Treg) play a critical role in regulating immune responses, as well as in preventing autoimmune diseases and graft rejection. It is known that antigen-specific Treg are more potent than polyclonal Treg in suppressing pathogenic immune responses that cause autoimmunity and inflammation. However, difficulty in identifying and isolating a sufficient number of antigen-specific Treg has limited their use in research to elucidate the mechanisms underlying their regulatory function and their potential role in therapy.

**Methodology/Principal Findings:**

Using a novel class II MHC tetramer, we have isolated a population of CD4^+^ Foxp3^−^ T cells specific for the autoantigen glutamic acid decarboxylase p286–300 peptide (NR286 T cells) from diabetes-resistant non-obese resistant (NOR) mice. These Foxp3^−^ NR286 T cells functioned as Treg that were able to suppress target T cell proliferation in vitro and inhibit type 1 diabetes in animals. Unexpected results from mechanistic studies in vitro showed that their regulatory function was dependent on not only IFN-gamma and nitric oxide, but also on cell contact with target cells. In addition, separating NR286 Treg from target T cells in transwell assays abolished both production of NO and suppression of target T cells, regardless of whether IFN-γ was produced in cell cultures. Therefore, production of NO, not IFN-gamma, was cell contact dependent, suggesting that NO may function downstream of IFN-gamma in mediating regulatory function of NR286 Treg.

**Conclusions/Significance:**

These studies identified a unique population of autoantigen-specific Foxp3^−^ Treg that can exert their regulatory function dependent on not only IFN-γ and NO but also cell contact with target cells.

## Introduction

Inflammation plays a central role during the development of autoimmune diseases. It is known that CD4^+^ regulatory T cells (Treg) are able to induce effective immune tolerance that inhibits such inflammatory responses [1], [2], [3], [4]. Defective development or depletion of Treg in animals, such as the naturally arising Foxp3^+^CD4^+^CD25^+^ Treg (nTreg), results in the development of autoimmune diseases, including type 1 diabetes (T1D) [1], [2], [3], [4], [5], [6], [7]. Administration of Treg, expanded *in vitro*, to animals has been shown to inhibit autoimmune diseases, including T1D [8], [9]. Previous studies have demonstrated that different cell populations with varied phenotypes can function as Treg, regulating both immunity and autoimmunity [10], [11], [12], [13], [14]. Both Foxp3^+^ and Foxp3^−^ Treg may play a pivotal role in the negative regulation of immune system function and inhibition of inflammation, as well as in the prevention of autoimmune diseases and graft rejection. Therefore, the use of Treg as a cellular therapy to induce immune tolerance in animals or humans may be a promising method to control inflammation and autoimmune diseases.

In addition to the heterogeneous nTreg, whose antigen-specificity remains largely unknown, our studies and those of others have demonstrated that cytokine-dependent antigen-specific Treg can be expanded following treatment with antigens [12], [15], [16], [17], [18]. Unlike nTreg, whose function is cell-contact dependent [1], [2], [3], [11], [19], antigen-induced Foxp3^−^ Treg may exert their function through the production of cytokines such as IL-10 [4], [13], [14], [16], [18]. It has been well-established that antigen-specific Treg are more potent than polyclonal Treg in suppressing pathogenic immune responses that cause inflammation [8], [9], [20], [21]. However, difficulty in identifying and isolating a sufficient number of antigen-specific Treg has limited the ability of researchers to elucidate the mechanisms underlying their regulatory function and their potential role in therapy. In studies to address this difficulty, we have generated autoantigen-specific class II MHC I-Ag7 tetramers to isolate several autoantigen-specific Treg from non-obese diabetic (NOD) as well as non-obese resistant (NOR) mice [16], [17], [22], [23].

NOR mice are a diabetes-resistant recombinant congenic strain of NOD mice [24], [25]. While both strains express the same disease-associated I-Ag7 molecules, NOR mice are resistant to T1D. Although the mechanism underlying disease resistance remains largely unclear, it is likely that NOR mice have a relatively more robust regulatory T cell function than their NOD counterparts [24]. In addition, the manner in which Treg exert their regulatory function may differ in NOR and NOD mice [17]. In order to better understand the function of autoantigen-specific Treg from NOR mice, as well as their role in T1D development, we have isolated NOR mouse CD4^+^ T cells specific for an immunodominant p286 (a.a. p286–300) peptide of a major autoantigen, glutamic acid decarboxylase (GAD), involved in T1D. We report our results from studies on these novel GAD p286-specific T cells.

## Results

### GAD p286-Specific CD4^+^ T Cells Isolated from NOR Mice Produce Both IFN-γ and IL-10

**Figure 1:**
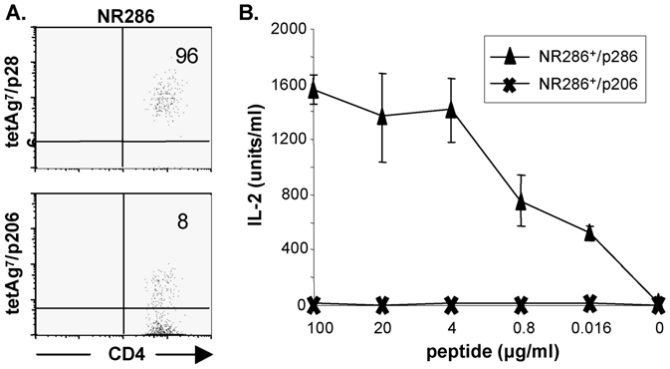
Isolation and characterization of CD4^+^ NR286 T cells. (**A**) FACS analyses of purified NR286 T cells stained with tetAg7/p286 tetramer and an anti-CD4 antibody or tetAg7/p206 (as a negative control). The numbers shown in each quadrant represent the percentage of cells stained by the tetramer. (**B**) IL-2 production by NR286 T cells (2×10^5^/well) in response to various concentrations of p286 or control p206 peptides plus NOR mouse APCs (4×10^5^/well). Peptides were 5-fold serially diluted from 100 µg/ml. HT-2 cells were used as the indicator in a MTT assay. Data represent average ± SEM, n = 3.

**Figure 2:**
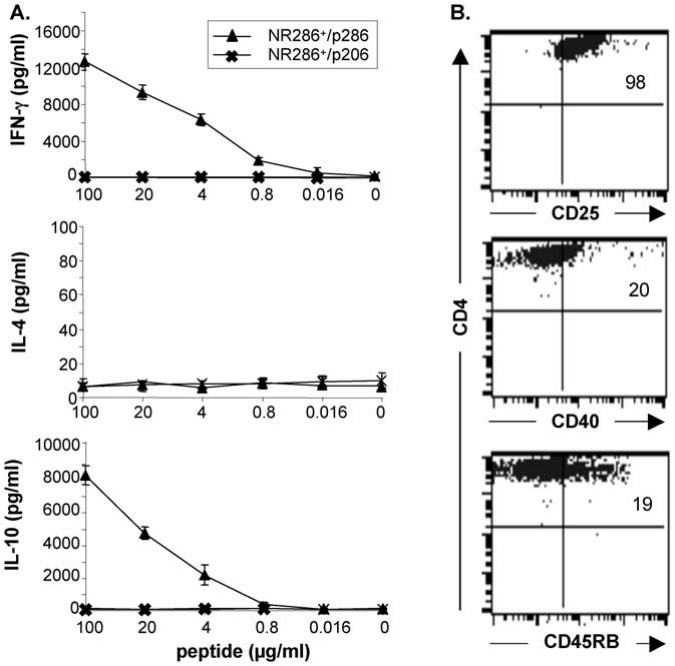
Cytokine production and phenotype analyses of NR286 T cells. (**A**) Analysis of cytokine production by NR286 T cells (2×10^5^/well) in response to various concentrations of p286 plus APCs. Cell culture supernatant was harvested after 24 h cell culture for ELISA (lower detection limit, 8 pg/ml for IL-4 and 30 pg/ml for IL-10 and IFN-γ). Data represent average ± SEM, n = 3. (**B**) Phenotypic analysis of NR286 T cells. NR286 T cells were co-stained with anti-CD4 plus antibodies against indicated markers. Numbers in each quadrant represent the percentage of cells positively detected by antibodies.

GAD p286-specific CD4^+^ T cell line (NR286 T cells) were isolated from p286-immunized NOR mice, using a novel I-Ag7 tetramer (tetAg7/p286) specific for this peptide, to investigate their function and potential role in T1D development. Our results suggested the established NR286 T cell line was p286-specific, as cells were positively stained by tetAg7/p286, but not by a control tetramer (tetAg7/p206) specific for a different GAD peptide, p206; additionally, they produced IL-2 in response to p286, but not to p206 (Fig. 1A and 1B). In studies to examine their response to antigen stimulation, ELISA results showed that NR286 T cells produced IFN-γ and IL-10, in a concentration-dependent manner, but not IL-4, in response to p286 stimulation (Fig. 2A). These results suggested that NR286 T cells may represent a novel population of T cells that are not only Th1-like, but also have acquired a Tr1-like cytokine secretion profile, producing both IFN-γ and IL10 [4], [13], [14].

Additional studies examined NR286 T cell phenotype, with results demonstrating that ∼98% of NR286 T cells expressed CD25 (but not Foxp3), ∼20% expressed CD40, and ∼19% expressed CD45RB (Fig. 2B). NR286 T cells did not express CD44, CD62L, CD69, or CTLA-4 (data not shown). These results suggested that although NR286 T cells produced IL-10, they were different from the Foxp3-expressing nTreg.

### NR286 T Cells Inhibited Diabetes Caused by NOD Mouse Splenocytes

**Figure 3:**
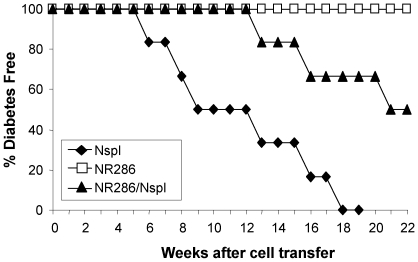
NR286 T cells inhibit diabetes development. NOD/scid recipient mice were transferred with either NOD mouse splenocytes (Nspl)(1×10^7^ cells/mouse) alone, NR286 T cells alone (1×10^7^ cells/mouse), or co-transferred with an equal numbers of both NR286 T cells and Nspl. The difference of diabetes onset and development between co-transferred mice and Nspl single transferred mice was significant (p<0.002).NOD mouse splenocytes were isolated from 7–8 wk old female NOD mice. At least 8 mice were studied in each group.

Because NR286 T cells produced both IFN−γ and IL-10, we examined whether they functioned as pro-inflammatory T cells or as Treg. Adoptive transfer experiments were performed to determine the *in vivo* role of NR286 T cells during diabetes development. NR286 T cells were adoptively transferred, either alone or with female NOD mouse splenocytes, into NOD/scid recipient mice. NOD mouse splenocytes were also transferred alone as controls. Recipient mice transferred with NOD mouse splenocytes alone developed diabetes as early as at 6 wk after the cell transfer, and all recipient mice became diabetic by 18 wk after cell transfer (Fig. 3). In comparison, transfer of NR286 T cells alone did not induce diabetes, suggesting they were not diabetogenic. Interestingly, NR286 T cells not only delayed diabetes onset by 7 wk, but also inhibited diabetes development when they were co-transferred with NOD mouse splenocytes (Fig. 3), as 50% of NOD/scid recipient mice that were co-transferred with NR286 T cells and NOD mouse splenocytes remained diabetes-free by the end of the experiment (22 wk after the cell transfer).

In summary, these *in vivo* studies demonstrated that NR286 T cells do not act like pro-inflammatory T cells that induce diabetes; instead, they may function as Treg, with the potential to inhibit diabetes development.

### NR286 T Cells Suppressed *In Vitro* Proliferation of Pathogenic BDC2.5 T Cells

**Figure 4:**
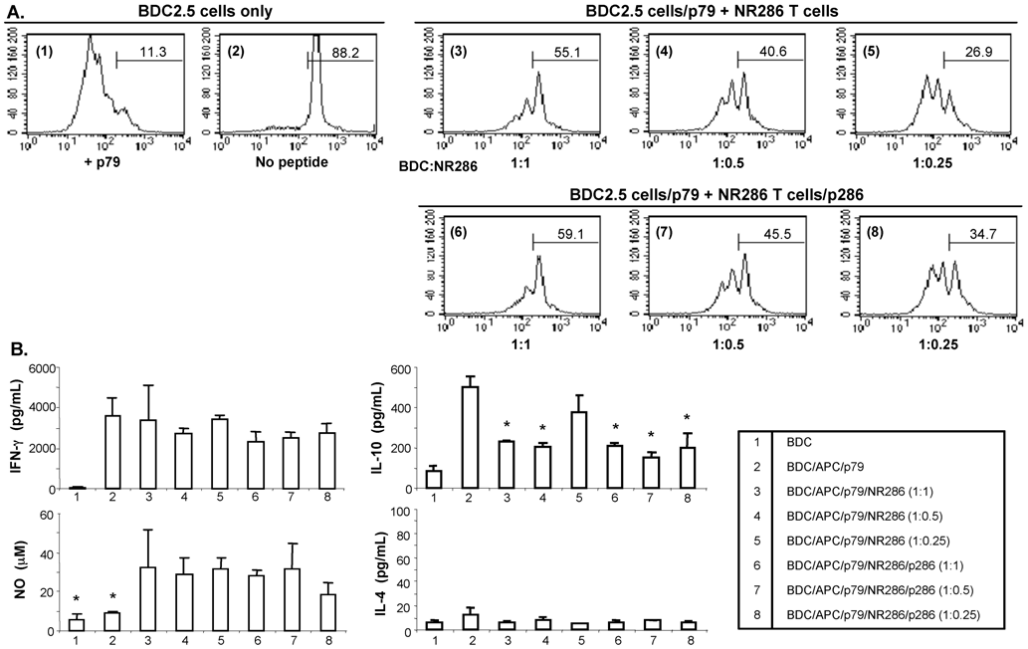
NR286 T cells suppress proliferation of BDC2.5 T cells. (**A**) Inhibition of CFSE-labeled BDC2.5 T cell proliferation co-cultured with NR286 T cells. CD4^+^ BDC2.5 T cells were isolated from BDC2.5 TCR transgenic mice and labeled with CFSE (2×10^5^/well), then cultured under eight different conditions (indicated as the numbers in parenthesis shown in each histogram). In control cultures, BDC2.5 T cells were cultured alone either (1) without p79 or (2) with p79 (0.1 µg/ml) plus CD4^+^ cell-depleted irradiated APCs from NOD mice. Cells also were co-cultured in the presence of either NR286 T cells (conditions 3–5) or with NR286 T cells plus p286 (conditions 6–8) for 4 days. NR286 T cells were added to cell cultures with a 2-fold serial dilution starting at a 1∶1 ratio (BDC2.5 T cells: NR286 T cells). The numbers shown in each histogram represent the percentage of cells that underwent <2 rounds of cell division. Data are representative from at least n = 3. (**B**) Cell culture supernatant was harvested from 4-day cell cultures under the same eight different conditions as shown in (**A**). Levels of IL-10 detected in conditions 3, 4, and 6–8 were significantly reduced compared to condition 2 (p<0.01). NO production in conditions 3–8 was significantly increased compared to conditions 1 and 2.

*In vitro* experiments were then performed under eight different culture conditions to further determine whether NR286 T cells functioned as Treg to suppress antigen-specific proliferation of other T cells. The proliferation of CFSE-labeled CD4^+^ BDC2.5 T cells in response to stimulation by p79, a highly active synthetic peptide in stimulating the diabetogenic BDC2.5 T cells [26], was measured with and without the presence of NR286 T cells in the culture. The results showed that only ∼11% of BDC2.5 T cells did not proliferate beyond more than two rounds of cell division in the presence of p79 (condition 1), compared to ∼88% in the absence of p79 (condition 2) (Fig. 4A). Our results further showed that both non-activated (Fig. 4A, conditions 3–5) and p286-activated NR286 T cells (conditions 6–8) were able to suppress the proliferation of BDC2.5 T cells in response to p79. The percentage of non-dividing CFSE-labeled CD4^+^ BDC2.5 cells and cells with less than two rounds of cell division increased from ∼11% to ∼55% in the presence of an equal number of non-activated NR286 T cells (condition 3), and to ∼59% in the presence of p286-activated NR286 T cells (condition 6). In addition, the suppression of BDC2.5 cell proliferation by NR286 T cells was concentration-dependent, that is, proportional to the number of NR286 T cells present in the cell culture containing BDC2.5 cells. The data showed that, compared to the 1∶1 BDC2.5:NR286 T cell ratio, the percentage of cells with<2 rounds of cell division at 1∶0.25 ratio decreased from ∼55% to ∼27% (condition 3 vs. 5; without p286 to activate NR286 T cells) and from ∼59% to ∼35% (condition 6 vs. 8; with p286 to activate NR286 T cells) (Fig. 4A).

In addition to measuring the suppressive effect of NR286 T cells on BDC2.5 T cell proliferation, cytokine production under the different culture conditions was also analyzed. ELISA was used to analyze supernatants collected from cell cultures under the various conditions with or without p286-activation of NR286 T cells. As predicted, the supernatant from all conditions, except BDC2.5 T cells alone, contained a comparative amount of IFN-γ (Fig. 4B). None of the cultures produced detectable levels of IL-4. In addition, compared to cultures containing BDC2.5 T cells activated by p79 plus antigen presenting cells (APCs)(Fig. 4B, condition 2), the production of IL-10 was significantly reduced in the presence of either p286-activated or non-activated NR286 T cells, especially when NR286 T cells were present at a ratio of 1∶1 or 1∶0.5 (Fig. 4B, conditions 3, 4, 6 and 7). While the reason for this reduction has not yet been identified, it could simply have been that the IL-10 produced by BDC2.5 T cells was suppressed by NR286 T cells. We were unexpectedly also able to detect a significant amount of nitric oxide (NO) present in the supernatants in all cell cultures containing NR286 T cells (Fig. 4B, conditions 3–8). In comparison, the supernatant from cell cultures containing BDC2.5 T cells alone, either with or without p79, did not produce significant amounts of NO.

These results demonstrated that NR286 T cells were able to function as Treg *in vitro* by suppressing the target T cell proliferation; soluble factors other than IL-4 may be involved in this suppressive process.

### The Regulatory Function of NR286 T Cells is Dependent on Production of Both IFN−γ and NO

**Figure 5:**
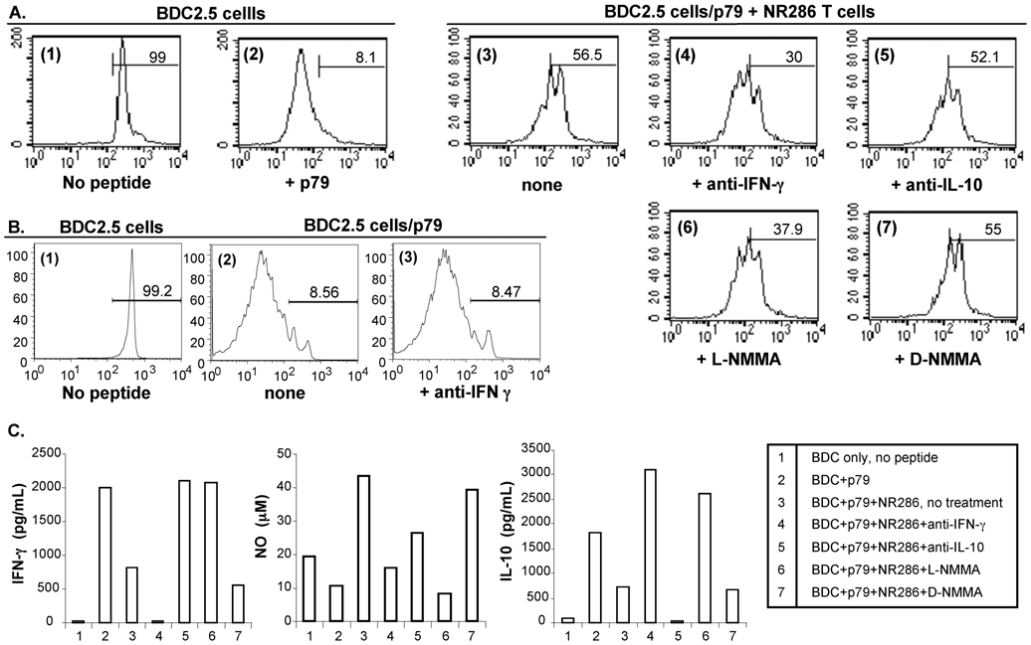
Blockade of IFN-γ and NO production abolished NR286 T cell regulatory function. (**A**) CFSE-labeled CD4^+^ BDC2.5 T cells (2×10^5^/well) were cultured under seven different culture conditions. In control cell cultures, BDC2.5 T cells were cultured in conditions of either (1) alone or (2) with p79 plus CD4^+^ cell-depleted irradiated APCs for activation. BDC2.5 T cells were also cultured with (3) an equal number of NR286 T cells. In some experiments, antibodies against (4) IFN-γ or (5) IL-10 (24 µg/ml), and NO inhibitors, either (6) L-NMMA (active) or (7) D-NMMA (inactive) (0.2 µM), were added to the cultures to determine their effect on blocking the regulatory function of NR286 T cells. The number shown in each histogram represents the percentage of BDC2.5 T cells that underwent <2 rounds of cell division. Data are from at least 2 experiments. (**B**) CFSE-labeled CD4^+^ BDC2.5 T cells (2×10^5^/well) were cultured under three conditions. Similar to those in (A), in control cell cultures, BDC2.5 T cells were cultured in conditions of either (1) alone or (2) with p79 plus CD4^+^ cell-depleted irradiated APCs for activation. BDC2.5 T cells were also cultured with (3) antibody against IFN-γ (24 µg/ml), in the absence of NR286 T cells. **(C)** Cell culture supernatants from (A) were harvested for ELISA to detect the presence of IFN-γ, IL-10, and NO.

In order to further understand how NR286 T cells may function as Treg, we investigated the mechanisms underlying their suppression of BDC2.5 T cell proliferation. In particular, cytokine or NO blockade experiments were performed under seven different culture conditions to examine whether NR286 T cell regulatory function was dependent upon secretion of soluble factors detected in cell cultures (Fig. 5A). Neutralizing antibodies against either IFN−γ or IL-10, or inhibitors against NO were added to the cell cultures to block the potential effect of these soluble factors on BDC2.5 T cell proliferation in the presence of NR286 T cells. The results showed that blockade of IFN-γ and NO, but not IL-10, resulted in a significant increase in BDC2.5 T cell proliferation. In the presence of an anti-IFN−γ antibody, the percentage of CFSE-labeled CD4^+^ BDC2.5 T cells with less <2 rounds of cell division decreased from ∼57% to 30% (Fig. 5A, condition 3). In control experiments, our results showed that anti-IFN−γ blockade alone did not alter BDC2.5 T cell proliferation (Fig. 5B, condition 2 vs. 3,). Therefore, these results excluded the possibility that anti-IFN−γ blocked IFN−γ production by BDC2.5 T cells, thereby enhancing its proliferation in the absence of NR286 Treg. Similarly, in the presence of the active NO inhibitor (L-NMMA), the percentage of non-dividing (or with reduced number of cell divisions) CD4^+^ BDC2.5 T cells also decreased to ∼38% (Fig. 5A, condition 6). On the other hand, the addition of an anti-IL10 antibody or an inactive NO inhibitor analogue (D-NMMA) to the cell cultures did not restore BDC2.5 T cell proliferation (Fig. 5A, conditions 5 and 7, respectively). These results suggested that NR286 T cell regulatory function was dependent on IFN−γ and NO production.

Additional mechanistic studies were then performed to better understand the relative inhibitory effects on production of individual soluble factors (IFN−γ vs. NO). Cell culture supernatants were collected from seven different culture conditions for further analyses. These results showed that NR286 T cells significantly reduced the production of IFN−γ in cell culture (Fig. 5C, conditions 2 vs. 3), while the production of NO was increased (Fig. 5C, conditions 2 vs. 3). In the presence of an anti-IFN-γ antibody (Fig. 5C, condition 4), IFN-γ was not detected in the cell culture supernatant. Unexpectedly, NO production (15 µM) in these cultures (Fig. 5C, condition 4) was also reduced compared to cultures without an anti-IFN−γ antibody (condition 3), which contained more than 40 µM of NO despite a reduced amount of their IFN−γ. These results suggested that blockade of IFN−γ production also suppressed NO production. In addition, although more IFN−γ was detected in cultures of p79-activated BDC2.5 cells without NR286 T cells (Fig. 5C, condition 2) than with NR286 T cells (condition 3), it did not lead to an increased NO production. These results suggested that IFN−γ production alone would not induce NO production in the absence of NR286 T cells.

In contrast to the IFN−γ blockade effect on NO production, addition of the active NO inhibitor, L-NMMA, to cell cultures not only inhibited NO production in cell cultures but also restored IFN−γ production (Fig. 5C, condition 6 vs. 3). As a negative control, the presence of the inactive NO inhibitor, D-NMMA, did not change the production of either IFN−γ or NO (Fig. 5C, condition 7 vs. 3). In addition, although blockade of IL-10 restored IFN−γ production, it also reduced NO production (Fig. 5C, condition 5 vs. 3).

Altogether, these results implicated IFN-γ and NO secretion as mediators of NR286 T cell regulatory function. In addition, these data suggested the production of IFN-γ may precede that of NO and help trigger NO production, resulting in suppression of the target BDC2.5 T cells.

### *In Vitro* Regulatory Function of NR286 T Cells Is Cell-Contact Dependent

**Figure 6:**
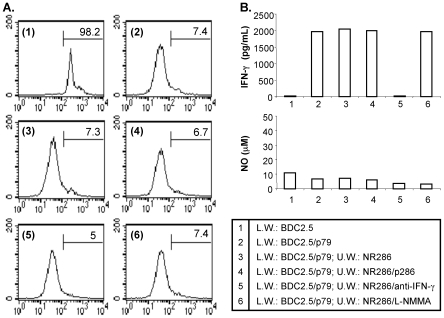
Inhibition of BDC2.5 T cell proliferation is cell contact dependent. (**A**) Transwell analyses of BDC2.5 T cell proliferation. CD4^+^ BDC2.5 T cells were isolated from BDC2.5 TCR transgenic mice and labeled with CFSE (5×10^5^/well). BDC2.5 T cells were cultured either (1) alone or (2) activated with p79 in the transwell lower well (L.W.). In the upper wells (U.W.), an equal number of (3) NR286 T cells alone, (4) NR286 T cells plus p286, or NR286 T cells in the presence of either (5) anti-IFN-γ or (6) the active NO inhibitor L-NMMA were cultured for 4 days. The CFSE-labeled BDC2.5 T cells (L.W.) were harvested and cell proliferation analyzed by FACS. Data are from at least 2 experiments. (**B**) Cell culture supernatants obtained from the six cell culture conditions were harvested for analysis of IFN-γ and NO production.

In order to examine whether NR286 T cell regulatory function depended solely on cytokine production, transwell assays were performed to determine whether NR286 T cells were still able to suppress cell proliferation without being in direct contact with the target BDC2.5 T cells. In these assays, NR286 T cells were cultured in the upper well and were separated from BDC2.5 T cells (cultured in the lower well) of transwell plates. CD4^+^ BDC2.5 T cells were labeled with CFSE and cultured either alone or stimulated with p79 in the presence of CD4^+^ cell-depleted APCs. Unexpectedly, neither p286-activated nor non-activated NR286 T cells suppressed BDC2.5 T cell proliferation (Fig. 6A, conditions 3 and 4 vs. condition 2). The addition of an anti-IFN-γ antibody or the NO inhibitor L-NMMA had no effect on either restoring or enhancing proliferation of BDC2.5 T cells (Fig. 6A, conditions 5 and 6).

IFN-γ and NO production were also assessed under the same culture conditions. IFN-γ was detected in the supernatant of cell cultures containing BDC2.5 T cells stimulated with p79 either in the absence or presence of NR286 T cells (Fig. 6B, conditions 2, 3, and 4). As expected, addition of an anti-IFN-γ antibody blocked the production of IFN-γ in cell cultures, and the NO active inhibitor L-NMMA had no effect on IFN−γ production (Fig. 6B, condition 5 and 6). In comparison, no significant levels of NO were detected in any of the conditions tested (Fig. 6B). These results suggested that cell contact between NR286 T cells and target cells was necessary for NO but not IFN-γ production and for suppression of BDC2.5 T cell proliferation.

Our data demonstrated that NR286 T cell regulatory function was not only dependent on IFN-γ and NO, but also on cell contact with target cells; similarly, NO production was also dependent on cell contact. These results provide evidence to support the conclusion that NO may be an effector molecule downstream of IFN−γ in mediating NR286 T cell regulatory function, which is also dependent on cell contact between NR286 T and target cells.

## Discussion

We report here a novel population of Foxp3-negative GAD p286-specific NR286 Treg isolated from NOR mice. While Th1-like NR286 T cells secreted significant amounts of IFN-γ, they functioned as Treg. Their regulatory function was dependent not only on IFN-γ, but also on NO and cell-contact with target cells. Similar to Foxp3-expressing CD4^+^CD25^+^ nTreg, NR286 Treg expressed CD25 and were cell-contact dependent; however, they did not express Foxp3. In addition, separating NR286 Treg cells from target T cells abolished both NO production and suppression of target T cells, regardless of whether IFN−γ was produced in cell cultures. These results showed that production of IFN−γ alone by antigen-activated pathogenic T cells, such as BDC2.5 T, was insufficient to induce NO production unless Treg, such as NR286 T cells, were also present.

Our studies did not distinguish the producers of IL-10, IFN−γ or NO in cell culture studies showing the suppression of BDC2.5 T cell proliferation by NR286 Treg. However, our results demonstrated that suppression of BDC2.5 T cells occurred only when NR286 Treg were present in cell cultures. In addition, blockade of IFN−γ alone, in the presence of NR286 Treg, did not alter BDC2.5 T cell proliferation. Even if these soluble factors produced by activated BDC2.5 T cells contributed to the suppression of BDC2.5 T cells, the presence of NR286 Treg was required for the suppressive effect. Also, the addition of p286 peptide to the culture medium had no effects on either the proliferation of BDC2.5 T cells or the production of IL-10 and IFN−γ, suggesting that the regulatory function of NR286 Treg was independent of their activation status. Our studies further showed that, in addition to soluble factors, suppression of BDC2.5 T cells was also dependent on cell contact between NR286 Treg and target cells; that is, the production of these soluble factors in cell cultures, in the absence of NR286 Treg, was insufficient to suppress BDC2.5 T cells.

The GAD p286 peptide, similar to p206 and p221 peptides, has been demonstrated as a major immunogenic peptide in NOD mice [27]. Our previous studies showed that GAD peptide-specific CD4^+^ T cells are able to function as Treg [16], [17]. Consistent with our results, other studies also demonstrated that GAD-specific CD4^+^ T cells from NOD mice, or CD4^+^ T cells from transgenic mice (expressing TCRs derived from NOD mouse T cell hybridomas specific for GAD peptides) inhibited T1D [28],[29]. In addition, our current results further demonstrated that p286-specific T cells isolated from NOR mice, like GAD-specific T cells from NOD mice, functioned as Treg. However, the mechanisms underlying NR286 Treg regulatory function appeared to be different from GAD-specific T cells isolated from NOD mice, as they were dependent on IL-10 and independent of cell contact with target cells [16], [18], [28], [29]. Altogether, these results showed that, regardless of the mechanisms underlying their regulatory function, potent diabetes-inhibitory autoantigen-specific Treg can be relatively easily induced *in vivo* using GAD peptides. Because the amino acid sequences of these GAD peptides are identical in both human and mouse, these results suggest the possibility their use to induce potent Treg as a therapy for treatment of T1D. This is further supported by recent phase II clinical trials showing that GAD-treated T1D patients showed expansion of Treg and enhanced preservation of islet cell mass [30].

NOR mice are a class II MHC I-Ag7-bearing recombinant congenic strain of NOD mice. However, unlike NOD mice, NOR mice do not develop diabetes, perhaps due to having a more robust Treg population [24], [25]. Our results suggest that the mechanisms underlying how GAD-specific Treg cells function may also differ between GAD-specific Treg isolated from these two different mouse strains. NOD mouse Treg, specific for GAD peptides, were IL-10-dependent and cell contact-independent, while NOR mouse GAD-specific Treg were IFN-γ- and NO-dependent as well as cell-contact dependent [16], [17], [18], [23]. The reason for this difference is currently unclear. One possibility could be due to the culture conditions used to derive NR286 T cells. However, we consider this unlikely given that the same cell culture protocol was previously used by us to isolate T cells specific for different GAD peptides (p206 and p221) as well as for an unrelated peptide (a mimetic epitope, p79, that is highly active in stimulating BDC2.5 T cells). Isolated T cells either functioned as Treg or as non-pathogenic cells [16], [17], [18], [23], and did not lead to a biased production of NR286-like Treg. An alternative possibility is that the difference in non-class II MHC genes in NOR and NOD mice may contribute to the functional difference between NOD and NOR mouse Treg. It is possible that non-class II MHC background genes can influence the selection of Treg with varied underlying regulatory mechanisms in either NOD or NOR mice. Further studies are necessary to elucidate the potential mechanisms responsible for the observed differences in the regulatory mechanisms underlying different Treg in these mice.

Although NR286 T cell regulatory function was dependent on cell-contact with target cells, their unique requirement of IFN−γ and NO for their regulatory functions and the fact that they did not express Foxp3 distinguishes them from Foxp3-expressing nTreg. In addition, production of NO, not IFN−γ, was also dependent on cell contact between NR286 T cells and target cells. It is likely that a combined regulatory effect of IFN-γ and NO may be directly involved in mediating suppression of target cells and inhibition of diabetes by NR286 Treg cells. The role of IFN−γ in the immune response is complex [31]. Its pathogenic role as a Th1 cytokine in autoimmune disease development has been well documented [31]–[35]. However, IFN−γ can also negatively regulate immune responses and contribute to immune regulation processes [31], [36], [37]. The reason why IFN-γ plays these dual roles in the immune response is unclear. It is possible that IFN-γ produced by pathogenic Th1 cells plays a pathogenic role in the absence of NR286 Treg [38], [39]. On the other hand, in the presence of Treg, such as NR286 T cells, IFN-γ triggers NO production, which is dependent on cell-contact between Treg and target cells. The resulting NO may then serve as the effector molecule that suppresses pathogenic T cells and regulates autoimmune diseases. Alternatively, IFN-γ may negatively affect the function of pro-inflammatory Th17 cells and, together with NO, suppress inflammatory responses [40]. Previous studies have demonstrated that NO, which can be produced by macrophages or dendritic cells, inhibits T cell proliferation [41], [42], [43]. It is possible that the recruitment of autoantigen-specific Treg, such as NR286 Treg, into an inflamed site, may facilitate the interaction between Treg and APCs at the site, and that the production of IFN−γ and NO results in the suppression of autoimmune responses.

In summary, the current studies have 1) identified a unique population of autoantigen-specific Treg (NR286 T cells) from a disease resistant strain of mice that can effectively suppress the proliferation of pathogenic T cells and inhibit T1D, 2) demonstrated that NR286 T cell function was dependent not only on the production of soluble factors, including IFN−γ and NO, but also on cell contact with target cells, leading to the suppression of pathogenic T cell proliferation and diabetes development, and 3) provided evidence to support the hypothesis that NOR mice contain a novel population of GAD-specific Treg that could help prevent these mice from diabetes.

## Materials and Methods

### Mice

NOR mice were purchased from the Jackson Laboratory (Bar Harbor, ME). BDC2.5 transgenic mice were a gift from Drs. D. Mathis and C. Benoist (Joslin Diabetes Center/Harvard Medical School, Boston, MA). All animals were housed in a specific pathogen-free environment and were used at 7–8 wk of age.

### Peptides and Immunization

GAD65 p286 (p286–300) and BDC2.5 T cell-stimulating peptide p79 (1040–79)[23],[26] were synthesized at the Beckman Research Institute, City of Hope and purified using reverse-phase HPLC to a purity of >90%. Animals were immunized i.p. with 100 µg of peptide emulsified in an equal volume of incomplete Freund's adjuvant (IFA)(Sigma, St. Louis, MO) on days 0 and 7. Spleens were removed from animals on day 14 for further experiments.

### Production of Class II MHC Tetramers

Production and initial use of the I-Ag7 tetramers specific for either GAD peptides or p79 have been previously described [16], [22], [23].

### Isolation and Staining of Tetramer^+^ T Cells and Cytokine Assays

The procedures have been previously described [16], [17], [18]. Briefly, splenocytes isolated from p286-immunized NOR mice were pooled and cultured in Click's medium (Life Technologies, Grand Island, NY) with peptide for 3 days. Cells were further incubated in RPMI medium supplemented with 5% fetal bovine serum and IL-2 before being separated into tetramer^+^ and tetramer^−^ cells. CD4^+^, tetramer^+^ T cells were isolated using FACS and magnetic beads (Miltenyi Biotech, Auburn CA). Purified tetramer^+^ cells were maintained in complete medium (CM: RPMI medium supplemented with 5% FBS, plus IL-2). For longer cell cultures, T cells were restimulated with peptide and irradiated APCs (3,000 rads).

Cell staining using tetramer has been previously described [16], [22], [23]. Briefly, T cells were stained with PE-labeled tetramer plus the anti-TCR β chain antibody, H57, (37^o^C, 1–2 h) and analyzed by FACS using FACSCaliber (Becton-Dickinson, San Jose, CA). All antibodies used for FACS and for *in vitro* inhibition assays were purchased from eBioscience or B-D Pharmingen (San Diego, CA).

The IL-2 bioassay has been previously described [16], [22], [23]. For ELISA, cell culture supernatant was harvested after incubating cells with peptides for 24 h. Capture ELISA (B-D PharMingen) was used, according to the manufacturer's instruction, to measure the amount of cytokines produced in the cell culture.

### Adoptive Transfer of T Cells into NOD/scid Mice

For adoptive transfer experiments, NOD/scid mice received a single i.v. injection of NR286 T cells (1×10^7^/mouse), NR286 T cells plus an equal number of NOD mouse splenocytes, or NOD mouse splenocytes alone. Recipient mice were monitored for up to 22 wk after cell transfer (when mice were 30 wk old) and were considered diabetic after two consecutive weeks of glycosuria ≥2% and blood glucose level ≥250 mg/dL.

### Assessment of NO Levels

NO production was measured using the Greiss assay [39]. Briefly, supernatant (100 µl) from cells cultured for 24 h or 4 days was added to 100 µl of a 1∶1 mixture of 1% sulfanilamide dihydrochloride in 5% H_3_PO_4_ buffer and 0.1% naphthylethylenediamine dihydrochloride (Sigma-Aldrich). The reaction was incubated (room temperature, 10 min) then measured at OD_550_ nm using an ELISA reader. Nitrate contents in the reaction were calculated using a sodium nitrite standard curve, with a detection threshold of 1 µM.

### *In Vitro* Inhibition Assays Using Co-Cultures

CD4^+^ T cells from BDC2.5 TCR transgenic mouse splenocytes were purified using magnetic beads and labeled with CFSE (carboxy-fluorescein diacetate succinimidyl ester). CD4^+^ T cells (1×10^7^ cells/ml) in serum-free PBS were incubated (37°C, 10 min) with CFSE (0.8 µM), washed, and used for further analyses.

CFSE-labeled CD4^+^ T cells from BDC2.5 mice (the target cells), cultured in the absence of NR286 T cells with or without p79 (0.1 µg/ml) were used as controls. CFSE-labeled cells were also cultured in the presence of NR286 T cells and p79, with or without GAD p286 (15 µg/ml). Irradiated (3,000 rad) CD4^+^ T cell-depleted NOD mouse splenocytes were used as the APCs. Cells were cultured in RPMI medium containing 5% FCS for 4 days, washed and stained with the tetAg7/p79 tetramer and an anti-CD4 antibody to identify BDC2.5 T cells. The effect of NR286 T cells on BDC2.5 T cell proliferation was analyzed using FACS to determine the intensity of the CFSE in the cells and whether the cell division was altered.

To determine the effect of different cytokines on target cell proliferation, a saturating amount of antibodies against IL-10 or IFN−γ (24 µg/ml) was added to cell cultures of *in vitro* inhibition assays; cells were incubated for 4 days before being harvested for analyses.

To determine the effect of NO on target cell proliferation, NO inhibitors were added to cell cultures, with procedures similar to those described above in the *in vitro* inhibition assays, except that a saturating amount (0.8 µM) of L-NMMA (an active NO inhibitor) or D-NMMA (an inactive NO inhibitor analogue) was added to cell culture.

### Transwell Assays

NOD mouse splenocytes (5×10^5^ cells/well) were cultured in the lower well of a 24-well transwell plate (Corning Inc., Corning, NY). NR286 T cells were cultured in the upper well in the presence of irradiated CD4^+^ T cell-depleted NOD mouse APCs. CFSE-labeled CD4^+^ BDC 2.5 T cells (5×10^5^ cells/well) were cultured in the lower well in the presence of irradiated APCs, with NR286 T cells cultured in the upper well. BDC2.5 T cells were activated with p79 (0.1 µg/ml). After 4 days, cells were washed and stained with tetAg7/p79 and an anti-CD4 antibody.

### Statistical Analyses

The Student's t test, Wilcoxon test, and χ2 test for statistical analyses were used to calculate the statistical significance for differences between experimental groups; p<0.05 was considered statistically significant.
